# Online and computer-assisted career guidance: Are students prepared for it?

**DOI:** 10.3389/fpsyg.2023.1117289

**Published:** 2023-03-22

**Authors:** Veronika Khurumova, Joana Carneiro Pinto

**Affiliations:** Catholic University of Portugal, Faculty of Human Sciences, Lisbon, Portugal

**Keywords:** careers office, online and computer-assisted career guidance, higher education students, career needs, career intervention

## Abstract

Career services should be a priority for any higher education institution. These structures should be prepared to support the design, implementation, and evaluation of academic and career plans and decisions, providing services properly aligned with students’ needs, preferences, and characteristics. The present study addressed these questions by analyzing data from an online survey administered to 361 Portuguese higher education students. Results indicate that most students do not seek support in these structures, despite having various needs, particularly in the scope of exploring the world of work, developing goals and implementing career strategies. Most students are open to the use of online and computer-assisted career guidance, although their preference is for in-person interventions. These results allow Career Offices to identify new directions and opportunities to work with students, while demonstrating their value to the academic community and the labor market.

## 1. Introduction

Unless further education is attained, the common goal of most (if not all) students upon graduation is employment. Over 80% of students claim that the subsequent employment after graduation is a critical factor in their decision to enter university (Stolzenberg et al., 2019). Therefore, under modern economic conditions, universities have found themselves involved in a competition in the developing market of educational services. Almost any higher educational institution nowadays faces the question of its students’ employability (e.g., Vinson et al., 2014; Crowne et al., 2020), and Career Offices, Career Services or Employability Centers have been established, in many universities worldwide, to support students with their career development and improve their competitiveness in the labor market.

Throughout the years, the scope of career guidance provided at universities reflected the changes in economic, political, social, generational, and cultural norms (Dey and Cruzvergara, 2014). In a VUCA (Volatile, Uncertain, Complex, and Ambiguous) environment, characterized by economic downturns, labor market changes (e.g., overall effect of technological advances and elimination of particular required skills; OECD, 2019), high levels of unemployment among college graduates (U.S. Department of Education, 2020), and, more recently, the impact of the COVID19 pandemic on the probable prevalence of remote jobs in the future (Bartik et al., 2020), it appears critical for universities to rethink the ways they help graduates’ transition into careers. Experts agree that nowadays the purposes of Career Offices should be more comprehensive to meet college students’ wide range of vocational concerns to better prepare them for a highly changing and competitive job market (Gallagher et al., 1992; Gallup, 2016). In order to help career offices develop, students’ specific requirements need to be assessed to provide an understanding of and fulfillment of their expectations.

However, finding out students’ career needs might not be enough on its own. Career offices are competing in the attention economy like everyone else, and in order to make students fully capitalize on the career services provided by their university, the right resources that maximize engagement are required. Thus, identifying what kind of specific types of career interventions students find useful and necessary is equally important to adapt the delivery (Hayden and Ledwith, 2014). Indeed, assessing students’ career needs and intervention preferences, and then aligning these expectations with the skills development at university career centers has a vital implication for all parties involved—universities, students, and future employers in the pursuit of a better workforce (e.g., Vinson et al., 2014; Crowne et al., 2020).

Several past studies have addressed the career needs assessment of university students on a national level in China (Li and Jung, 2021); a single-university level in the United States (Fouad et al., 2006) and Romania (Crişan et al., 2015); and, on a faculty level in Turkey (Güneri et al., 2016) and Taiwan (Yang and You, 2010). Moreover, overall counseling needs were explored in Finland (Lairio and Penttinen, 2006), Greece (Giovazolias et al., 2010), and Portugal (Pinto et al., 2016; Pinto, 2019). Results indicate that, with differences depending on the population, gender, age, race, and socioeconomic status, the needs for help in academic and professional topics seem to surpass the needs for personal and social support (Giovazolias et al., 2010). In general, most of the students search for and receive career counseling or guidance in the final years of their studies (Pereira et al., 2020). Commonly stated career needs include obtaining information about the world of work, the transition from university to work life (e.g., job search strategies, Pinto, 2019), career planning, and stress management (Güneri et al., 2016), as well as, overcoming procrastination strategies and time management skills (Pinto, 2019). Also, students are quite passive about using these services (Crişan et al., 2015), preferring the employers to come to the university and display open vacancies rather than having to seek the information themselves. Finally, students indicate a preference for face-to-face rather than online interventions and group interventions rather than individual (Crişan et al., 2015). Most students are not very satisfied with the services provided by their career offices (Pereira et al., 2020).

Indeed, researchers agree on the need for an accurate and regular assessment of college students’ needs (Gallagher et al., 1992), to maintain high standards of the provided services. But, despite the importance of developing the services of career offices and their alignment with the expectations of students, to increase the competitiveness and employability of graduates, it appears that not all higher education institutions in Portugal have either fully operating career centers or developed and accessible sets of services provided (Khurumova, 2022). With a significant annual growth of the number of tertiary education students in the country, both local and foreign ones^1^ and paradoxically high level of youth unemployment (18.2% as of the third quarter in 2019, with an EU average of 14.4%)^2^, the provision of career services might be even more important in the country.

Considering the fact that the provision of face-to-face career guidance services is universally limited due to time allowance and personnel number (e.g., one counselor on average for 2,500 students at Master’s level according to NACE, 2017), online services seem beneficial in a sense they can be provided to a larger number of students and at their convenient timing. Nowadays, the Internet has steadily become an important aspect of everyone’s life regardless of age, as a source of communication, entertainment, and information search. Thus, as a result, this increasing use and reliance on Internet and technology has created opportunities for career counseling professionals to rethink and develop their services (Zainuddin et al., 2020). CAEL (2018) in its report noted several ways for reinventing career services and, in fact, incorporating technology to better assist students in engaging with career-related activities and connecting with employers is one of them. According to the report, technology enables for the provision of virtual career services and tools, target outreach to students, and new means of connecting with employers and business owners. Today, it is impossible to imagine effective university entities such as Career Offices that do not use Internet resources in their work. One advantage of technology-assisted or mediated career counseling is that it is available 24 h a day, 7 days a week, and provides instant feedback, which appeals to the younger generation (Zainuddin et al., 2020). Other advantages of online interventions may include the dynamism and less time needed to update information (Venable, 2011), possible interaction with employers (e.g., through incorporated social media; Venable, 2011), wider outreach (Zainuddin et al., 2020), and a vast number of methods to provide the guidance (Zainuddin et al., 2020). Nevertheless, it is very important, for all the above mentioned, to understand if these added values of online career and counseling are also understood in this way by its users (i.e., the students).

The present work involves an exploratory study aiming to identify and prioritize current career needs of students enrolled in Portuguese higher education. More specifically, it aims to understand how career offices should adapt in order to align with the needs of the service recipients and whether nowadays online interventions are of demand among students. Thus, the findings of the study contribute to the understanding of what subsequently could be used in the development of such interventions and/or to the adaptation of the existing services provided. This should result in a service that meets students’ needs more effectively.

## 2. Methods

### 2.1. Participants

A total of 361 students enrolled in 18 Portuguese Higher Education Institutions (HEI) in the academic year of 2020/2021 participated in this study (female = 281, 77.8%). Participants’ ages ranged from 17 to 60 years (*M*_age_ = 24, *SD* = 6.8). Majority of the sample were pursuing a bachelor’s degree (*n* = 170, 50%) and master’s degree (*n* = 171, 47%); PhD students only accounted for 3% of the sample (*n* = 11). They were pursuing their studies in Social and Behavioral Sciences (*n* = 221, 61.22%), Humanities, Law, or Educational Sciences (*n* = 86, 23.82%), Business Sciences (*n* = 22, 6.09%), Cultural and Artistic Programs (*n* = 15, 4.16%), Life Sciences and Mathematics (*n* = 10, 2.77%), Engineering (*n* = 5, 1.38%), and Medical Sciences (*n* = 2, 0.55%), enrolled either in public (*n* = 122, 34%) or private institutions (*n* = 239, 66%).

### 2.2. Instrument

*Career Offices in Higher Education: Needs Assessment Questionnaire* was specifically developed for this study, taking into consideration the Career Self-Management Model of Greenhaus et al. (2009) (Pinto and Taveira, 2010), which considers career management as a decision-making and problem-solving process that encompasses four main stages: self-knowledge; exploration of the environment; development of goals; and development and implementation of action plans. The development of the items also considered other previously existing questionnaires published in scientific research (e.g., McBride and Muffo, 1994; Briscoe, 2002; Yang and You, 2010; Güneri et al., 2016; Pinto, 2019). This questionnaire was submitted for (i) evaluation of the content validity with a group of experts; (ii) evaluation of the clarity of the questionnaire content, and completion of the questionnaire with a 30-day interval for calculation of the Intraclass Correlation Coefficient, with a group of students; and (iii) study of validity and internal consistency through Confirmatory Factor Analysis (Khurumova, 2022).

This instrument is organized as follows: (i) *Knowledge about the Careers Offices of your Higher Education Institution*: three questions that assess whether students are aware of the existence of a Career Office at their HEI, how they obtained this knowledge, and if they have ever used its services; (ii) *Preferred Career Intervention Modality:* a list of 10 options from which students select the preferred three in terms of career intervention. The different options include, for example, individual or group career counseling sessions, in-person or online career counseling sessions, employability workshops, and mentoring. There is also a question that allows students to indicate another preferred type of support not mentioned in the list; (iii) *Career Needs of Higher Education Students*: a list of 23 career needs, which include, for example, the need for support in preparing a CV (*curriculum vitae*), the need for support in using social platforms for job search, and the need for support in negotiating job offers. Responses to each item are made using a four-point Likert scale (1 = no need and 4 = high need); (iv) *Own Career Needs*: the previous list of 23 career needs, from which the students select the five that best represent their current support needs; (v) *Joining Online Career Services*: through a single item (0–10 points—Net Promoter Score) the probability of the students’ adherence to an online or computer-assisted career counseling service is assessed, if it were made available by their HEI; and (vi) *Other Additional Comments*: an open-ended question, in which students were asked to indicate any additional comments regarding their university’s Careers Office and the services it provides.

### 2.3. Data collection procedures

All research projects developed within the scope of the Faculty of Humanities (FCH) of the Catholic University of Portugal (UCP) had to be, at the time of the development of this study, submitted for approval to the Católica Research Center for Psychological, Family and Social Wellbeing (CRC-W). This submission implied a presentation of the study in terms of pertinence, theoretical basis, objectives, methodology, and procedures and ethical care in the data collection process (namely, questions regarding anonymity and confidentiality of data, withdrawal from participation without any penalty). Data were collected between January and August 2021. The Careers Office of the FCH-UCP sent by email an invitation to all students to collaborate in this research and the Office of Communication and Marketing (GCM) released it on various digital platforms. Moreover, each of the Careers Offices of a total of 89 Portuguese HEIs were also contacted *via* email or through their social platforms (e.g., Facebook page) with a request to distribute the questionnaire among their students. For this purpose, an Ethical Declaration from the CRC-W was submitted upon request. The invitation email contained information regarding the purpose of the study, as well as the link to the assessment protocol inserted in the Qualtrics platform.^3^ The assessment protocol included a more detailed explanation of the goal of the study, an informed consent, the assessment instrument previously presented, and a brief sociodemographic questionnaire. The average time to complete the assessment protocol was 8 min.

### 2.4. Data analysis procedures

Data were entered into a database and processed using software for statistical analysis (IBM SPSS Statistics for Windows, Version 26.0; IBM Corp, 2019). Exploratory data analyses were performed to examine if there were problems in the data such as outliers, non-normal distributions, problems with coding, and/or missing values, and to examine the extent to which the assumptions of the statistics that we planned to use were met. We used descriptive statistics to analyze the students’ needs and intervention preferences. In addition, correlational analyses between preferred career intervention modality and higher education students’ career needs were also performed. Also, several decision trees were conducted to predict the likelihood that students would use an online career service if it were provided by the Career Office of their respective universities/colleges. The decision tree procedure produces a tree-based classification model that arranges cases into groups or predicts values of a dependent (target) variable based on an independent variable (predictor; IBM SPSS Decision Trees 26). The decision trees were carried out using the CHAID method (chi-squared automatic interaction detector algorithm). The risk and classification tables were analyzed to provide an evaluation of how well the models work. All results were considered statistically significant yielding a value of p lower than 0.05 (*p* < 0.05).

## 3. Results

### 3.1. Knowledge about the career offices at HEI’s

Results indicate that most students, namely 277 participants (76.7%) heard about their Careers Offices, while 84 students (23.3%) did not. That information was mostly obtained through emails from the Careers Offices (*n* = 16, 4.7%); other common sources stated were *From other students* (*n* = 30, 8%), *Referred by lecturer* (*n* = 29, 8%), and *Institutional webpage* (*n* = 22, 6%). In terms of the Career Offices services usage frequency, out of those who knew about the Career Office, 263 (72.9%) never used its services, 74 (20.5%) and 15 (4.2%) used the services 1–2 or 3–4 times a year, respectively.

### 3.2. Preferred career intervention modality

The career intervention modalities that are most preferred are (see Table 1; highlighted in bold): *Online information about internship and/or job opportunities* (*n* = 230, 64%), *in-person individual guidance*, *and counseling sessions* (*n* = 187, 52%), *career mentoring programs/ sessions* (*n* = 185, 51%), *Career events* (*n* = 15, 44%), and *in-person workshops* (*n* = 147, 41%).

**Table 1:** Preferred career intervention modality: Frequencies and percentages.

Modality	Freq. (%)
In-person individual career guidance/counseling sessions	**187 (51.8%)**
In-person group career guidance/counseling sessions	62 (17.2%)
Online individual career guidance/counseling sessions	111 (30.7%)
Online group career guidance/counseling sessions	51 (14.1%)
In-person workshops (e.g., development of soft skills)	**147 (40.7%)**
Webinars (e.g., development of soft skills)	106 (29.4%)
Career mentoring	**185 (51.2%)**
Career events (e.g., career fairs)	**159 (44.0%)**
Printed information (e.g., about internships and job opportunities)	69 (19.1%)
Online information (e.g., about internships and job opportunities)	**230 (63.7%)**

### 3.3. Career needs of higher education students

Table 2 presents a list of 23 career needs for which the participants were asked to indicate, from their point of view, the degree of need of higher education students, on a scale of 1–4 points. Participants indicate as the main needs of students in higher education (highlighted in bold): *Identifying the information about possible employment opportunities and required qualifications* (*M* = 3.66, *SD* = 0.551), *Learning job search strategies* (*M* = 3.55, *SD* = 0.674), *Developing job interview skills* (*M* = 3.53, *SD* = 0.683), *Discussing career strategies that increase the likelihood of achieving my career goal* (*M* = 3.52, *SD* = 0.675), and *Gaining experience through internships* (*M* = 3.52, *SD* = 0.628).

**Table 2:** Career needs of higher education students.

Career needs	Mean (SD)
Determining own interests and developing new ones	2.99 (0.877)
Determining own skills and developing new ones	3.29 (0.734)
Determining own values and lifestyle	2.60 (1.047)
Determining personality traits and its relationship with specific environment contexts	2.86 (0.935)
Exploring different career options, such as determining my own choice between advanced studies or employment after graduation	**3.40 (0.750)**
Determining the paths to advanced studies in domestic and international programs	3.35 (0.767)
Identifying the type of job I’m best fitted for	**3.48 (0.715)**
Identifying the information about possible employment opportunities and required qualifications	**3.66 (0.551)**
Learning job search strategies	**3.55 (0.674)**
Developing clear, specific, and realistic career goals	3.38 (0.762)
Discussing career strategies that increase the likelihood of achieving my career goal	**3.52 (0.675)**
Selecting a new academic degree (e.g., Masters/PhD degree)	3.05 (0.822)
Applying for a scholarship	3.24 (0.798)
Gaining experience through internships	**3.52 (0.628)**
Writing cover letters	3.21 (0.802)
Preparing a résumé/CV	3.32 (0.835)
Developing job interview skills	**3.53 (0.683)**
Using social media platforms to search for job offers (e.g., LinkedIn)	3.13 (0.849)
Negotiating job offers	**3.50 (0.708)**
Networking effectively	3.47 (0.666)
Transferring skills gained in the course to the workplace	3.39 (0.738)
Learning how to be a freelancer or start my own business using knowledge gained from my degree	3.32 (0.786)
Supporting the soft-skills development (e.g., teamwork, communication, problem-solving, and leadership workshops)	3.29 (0.762)

### 3.4. Personal career needs

Table 3 presents a list of 23 career needs for which participants were asked to indicate which five they considered to best represent their own current support needs. The majority (*n* = 189, 52%) indicated that their priority in terms of current career needs was (highlighted in bold): *Identifying the type of job I’m best fitted for*; second most popular option (*n* = 133, 37%) was *Exploring different career options, such as determining my own choice between advanced studies or employment after graduation*, followed by *Gaining experience through internships* (*n* = 123, 34%). The lowest number of participants (*n* = 21, 6%) expressed the need for *Using social media platforms to search for job offers*.

**Table 3:** Personal career needs of higher education students.

Personal career needs	Freq. (%)
Determining own interests and developing new ones	65 (18%)
Determining own skills and developing new ones	94 (26%)
Determining own values and lifestyle	32 (8.9%)
Determining personality traits and its relationship with specific environment contexts	37 (10.2%)
Exploring different career options, such as determining my own choice between advanced studies or employment after graduation	**133 (36.8%)**
Determining the paths to advanced studies in domestic and international programs	53 (14.7%)
Identifying the type of job I’m best fitted for	**189 (52.4%)**
Identifying the information about possible employment opportunities and required qualifications	**105 (29.1%)**
Learning job search strategies	**101 (28%)**
Developing clear, specific and realistic career goals	76 (21.1%)
Discussing career strategies that increase the likelihood of achieving my career goal	101 (28%)
Selecting a new academic degree (e.g., Masters/PhD degree)	57 (15.8%)
Applying for a scholarship	43 (11.9%)
Gaining experience through internships	**123 (34.1%)**
Writing cover letters	35 (9.7%)
Preparing a résumé/CV	61 (16.9%)
Developing job interview skills	89 (24.7%)
Using social media platforms to search for job offers (e.g., LinkedIn)	21 (5.8%)
Negotiating job offers	63 (17.5%)
Networking effectively	64 (17.7%)
Transferring skills gained in the course to the workplace	66 (18.3%)
Learning how to be a freelancer or start my own business using knowledge gained from my degree	69 (19.1%)
Supporting the soft-skills development (e.g., teamwork, communication, problem-solving, and leadership workshops)	52 (14.4%)

### 3.5. Joining online career services

A mean score of 7.53 (*SD* = 2.197, *Min-Max* = 0–10) about the students’ likelihood of joining an online or computer-assisted career counseling service, if it were made available by their college, was found. Since a Net Promoter Score was used, 98 (27%) of the participants were classified as having a detractor attitude, 128 (36%) a passive attitude, and 135 (37%) a promoting attitude toward joining this type of service.

### 3.6. Career needs of higher education students and preferred career intervention modalities: Relationships exploration

The relationship between the career needs of higher education students and the preferred career intervention modalities was analyzed. There is a preference for in-person and individual career intervention modalities (*vs* online or group). More specifically, in-person individual career guidance is the preferred modality for *determining own interest and developing new ones* (*r* = 0.162, *p* = 0.002), *determining own skills and developing new ones* (*r* = 0.190, *p* ≤ 0.000), *determining own values and lifestyle* (*r* = 0.138, *p* = 0.009), *determining personality traits and its relationship with specific environment contexts* (*r* = 0.100, *p* = 0.043), *identifying the type of job I’m best fitted for* (*r* = 0.112, *p* = 0.034), *developing clear, specific and realistic career goals* (*r* = 0.110, *p* = 0.038), and *negotiating job offers* (*r* = 0.104, *p* = 0.040).

Also, in-person workshops are the preferred modality to *explore different career options such as determining my own choice between advanced studies or employment after graduation* (*r* = 0.126, *p* = 0.017), *learning job search strategies* (*r* = 0.176, *p* = 0.001), *discussing career strategies that increase the likelihood of achieving my career goal* (*r* = 0.119, *p* = 0.023), *selecting a new academic degree* (*r* = 0.108, *p* = 0.041), *gaining experience through internships* (*r* = 0.144, *p* = 0.006), *developing job interview skills* (*r* = 0.106, *p* = 0.044), *using social media platforms to search for a job* (*r* = 0.151, *p* = 0.004), *negotiating job offers* (*r* = 0.133, *p* = 0.011), *transferring skills gained in the course to the workplace* (*r* = 0.154, *p* = 0.003), and *supporting the soft skills development* (*r* = 0.147, *p* = 0.005). In-person group career guidance is also preferred to *learn how to be a freelance or start my own business* (*r* = 0.105, *p* = 0.046).

When the topic concerns *developing clear, specific and realistic career goals*, or *discussing career strategies that increase the likelihood of achieving my career goal*, students prefer online career guidance, individual (*r* = 0.133, *p* = 0.011) in the first case and in groups on the second (*r* = 0.115, *p* = 0.029). But, for both career needs, they also consider career mentoring (*r* = 0.130, *p* = 0.014; *r* = 0.112, *p* = 0.033). Career events are the preferred modality to *identify the information about possible employment opportunities and required qualifications* (*r* = 0.128, *p* = 0.015).

### 3.7. Probability of attending an online career guidance or intervention program

Next, we present the results of several decision trees conducted to predict the likelihood that students would use an online career service if it were provided by the Career Office of their respective universities/colleges. The dependent variable used, *If the Career Office at your school/university had an online and computer-assisted career guidance intervention or program: what would be the probability for you to attend it?*, since it is a Net Promoter Score type variable, was organized into three categories: students who are detractors, students who are passive, and students who are promoters of using these types of services. Three independent analyses were performed. In each of these analyses, the independent variables used were: (i) sociodemographic (gender, age, academic degree, public vs. private institution, area of study), (ii) the higher education students’ career needs, and (iii) own career needs.

#### 3.7.1. Decision tree with sociodemographic variables

**Figure 1:**
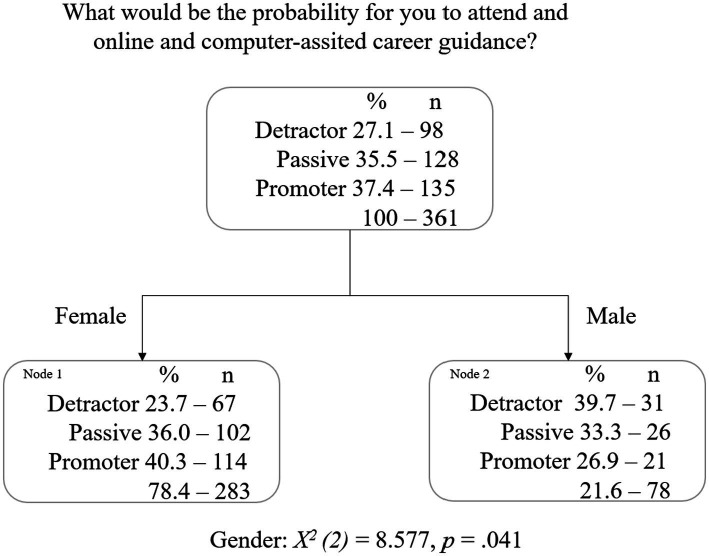
Attending an online career guidance or intervention program: Decision tree with sociodemographic variables.

This tree diagram (Figure 1) shows that gender is the best predictor of *what would be the probability for you to attend an online career guidance or intervention program* [*X^2^* (2) = 8.577, *p* = 0.041]. Female students are more likely to be promoters (40.3%) of this type of service (vs 36% passive and 23.7% detractor). In contrast, male students are more likely to be 39.7% detractors (vs 33.3% passive and 26.9% promoters).

The risk estimate of 0.598 (*error* = 0.026) indicates that the risk of misclassifying a student is approximately 60%. The model classifies approximately 40.2% of the students correctly. For those students with a passive behavior, the model predicts 0% of them—these are often classified as being promoters; for those students with a detractor behavior, it predicts their behavior in only 31.6%% of the cases, which means that 69% of students are inaccurately classified; and, for those students with a promotor behavior, the model predicts accurately 84.4% of them.

#### 3.7.2. Decision tree with the career needs of higher education students

**Figure 2:**
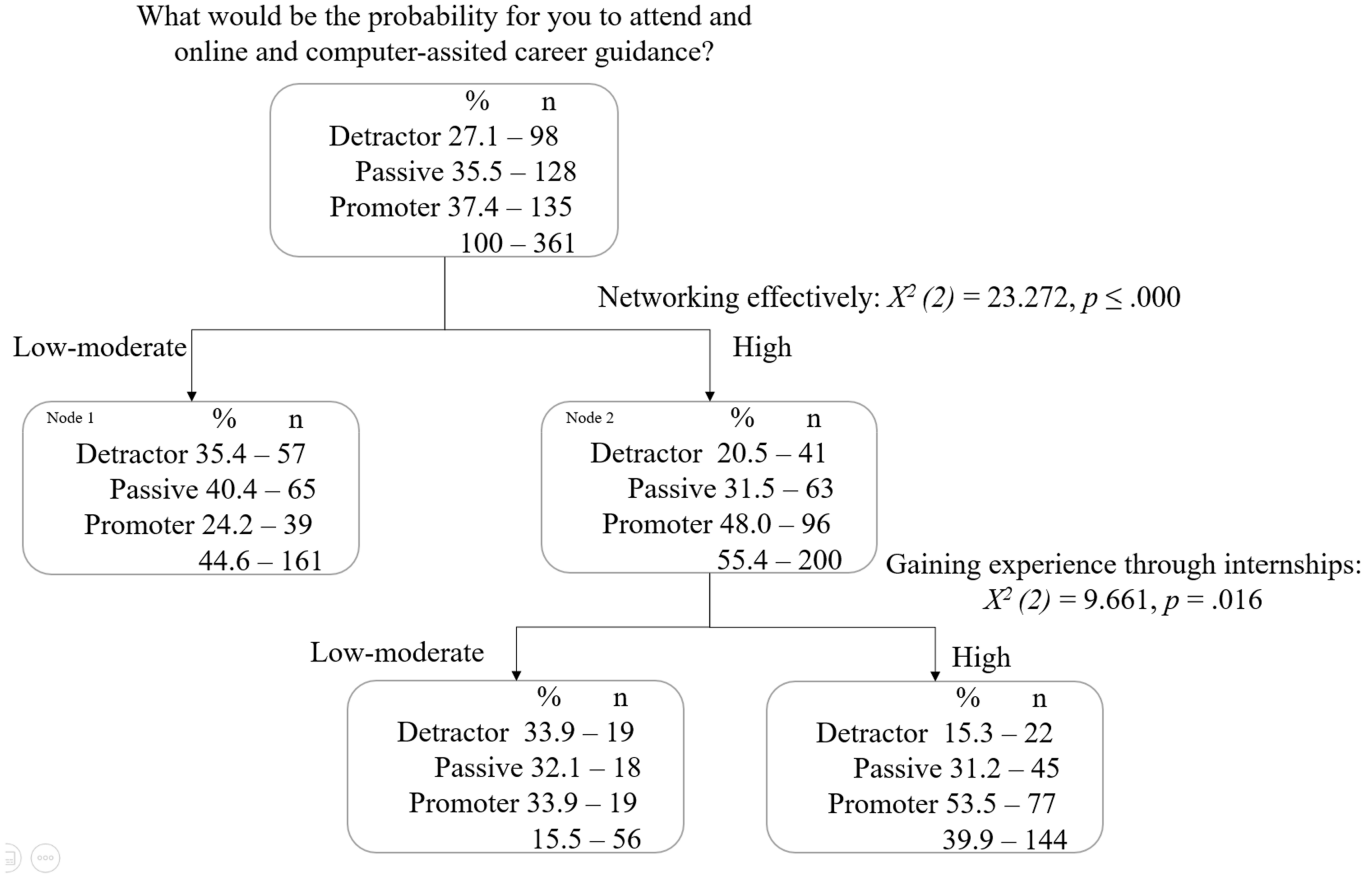
Attending an online career guidance or intervention program: Decision tree with career needs of higher education students.

This tree diagram (Figure 2) shows that the personal concern *networking effectively* is the best predictor of *what would be the probability for you to attend online career guidance or intervention program* [*X^2^* (2) = 23.272, *p* ≤ 0.000]. For the category of those with a reduced need, this is considered a terminal node since there are no child nodes. Within this category, 40.4% of students take a passive behavior on the likelihood of seeking online and computer-assisted career guidance for this topic, while 35.4% of students take a detractor behavior. For the category of students who have a moderate to high need, the model includes one more predictor—*gaining experience through internships*. Of the students who indicate a low need, 33.9% have a promoting behavior (vs 33.9% detractor and 32.1% passive) while of the students who indicate a moderate to high need, 53.5% have a promoting behavior (*vs* 15.3% detractor and 31.5% passive).

The risk estimate of 0.554 (*error* = 0.026) indicates the risk of misclassifying a student is approximately 55%, which means that the model classifies approximately 44.6% of the students correctly. Students with a passive behavior, are wrongly classified as promoters almost 50% of the times; and those with detractor behavior are never (0%) correctly identified. For those students with a promotor behavior, the model predicts accurately 71.1% of them.

#### 3.7.3. Decision tree with the variables of own career needs

**Figure 3:**
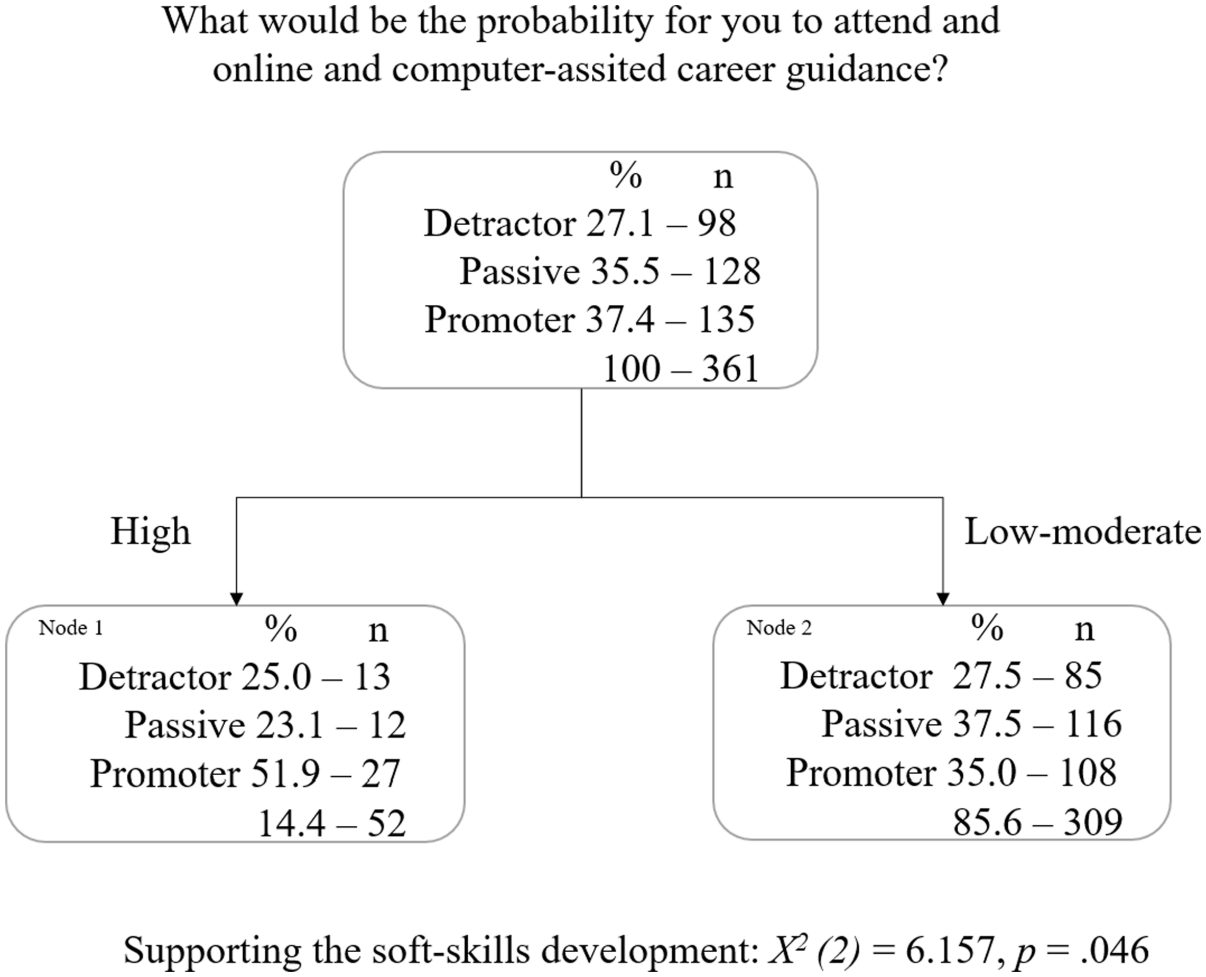
Attending an online career guidance or intervention program: Decision tree with own career needs.

This tree diagram (Figure 3) shows that the personal concern *supporting the soft-skills development* is the best predictor of *what would be the probability for you to attend an online career guidance or intervention program* [*X^2^* (2) = 6.157, *p* = 0.046]. For both groups, those who feel the need and those who do not feel the need to have soft skills development support, this is considered a terminal node, since there are no child nodes. However, considering those who do not feel this type of need, their behavior toward using an online career service is mostly passive (37.5%), while those who feel this type of need tend to adopt mostly a promoting behavior (51.9%).

The risk estimate of 0.604 indicates that the category predicted by the model is wrong for 60% of the cases (*error* = 0.026), which indicates that the model classifies approximately 39.6% of the students correctly. For those students with a detractor behavior, the model predicts correctly 0% of them; for those students with a promoter behavior, it predicts their promoting behavior in only 20% of the cases, which means that 80% of students with a promoter behavior are inaccurately classified with a passive behavior. But, students with a passive behavior are accurately identified in 90.6% of the cases.

## 4. Discussion

This paper focuses on a study aimed to identify current career needs of students enrolled in Portuguese higher education and whether online interventions would be of demand among those students. Based on the results of the analysis, several assumptions can be drawn.

Even though most students are aware of the existence of a career office at their university, only a small proportion of the participants have ever used its services. Compared to the study by Gallup (2016) that stated that 52% of students in the US tend to visit Career Offices at least once over their undergraduate studies, our findings are clearly lower than expected. Most students claim they need career support, yet, based on our findings they rarely apply for it. These results are congruent with those obtained in the study by Crişan et al. (2015), which indicate a passive attitude by most students about seeking support on career topics. Career offices should invest in more intensive and comprehensive marketing plans that bring them closer to their target audience.

Considering the career needs, it is important to analyze that the ones most highlighted by students are organized in two major areas: exploring the world of work and developing strategies that favor the achievement of career goals. Such services range (e.g., internship experiences and job vacancies; job interview preparation sessions) is quite common at career offices, and the majority of offices do provide job interview preparation sessions on demand and distribute information on internships and employment. What is interesting, however, is when asked about current personal needs, the results show that students need counseling support in understanding whether they should continue studying or finding employment after graduation and with the latter, identifying the type of employment that would be the best fit. But there is hardly any focus on self-knowledge, with aspects such as knowing one’s life values and personality traits being two of the least valued needs. Compared to previous studies, there is a tendency to focus on aspects such as job search strategies and career decision-making, devaluing the self-exploration dimension that should underpin the career management process (Yang and You, 2010; Crişan et al., 2015; Pinto, 2019). It therefore becomes necessary to raise student’ awareness of the importance of the topic of self-knowledge.

The results from correlation analysis allow to draw conclusions about which concerns students want to tackle using specific intervention types. It is clear that in-person individual career guidance sessions can be used to address several personal needs, such as determining and developing own interests, skills, values, and goals, whereas in-person workshops can be used for topics on learning job strategies, negotiating job offers, and developing soft skills.

Lastly, despite most students being interested in receiving online career information (e.g., regarding internships and job vacancies), the most preferred modalities of intervention are nevertheless the in-person. This is consistent with results from previous studies (e.g., Crişan et al., 2015; Pinto et al., 2016; Pinto, 2019), although in contrast to the study by Crişan et al. (2015), Portuguese students systematically indicate a preference for individual sessions or workshops over group counseling sessions (Pinto et al., 2016; Pinto, 2019). It is clear that in-person individual career guidance is preferred to address several self-exploration needs, such as determining and developing own interests, skills, values, and goals, whereas in-person workshops can be used for topics on learning job strategies, negotiating job offers, and developing soft skills.

In terms of possible on-line career interventions, most participants welcome such option, yet again, out of listed possible intervention modalities, online individual counseling sessions are the top preferences by respondents. This is congruent with prior research that states a possible need for human interaction (with a counselor) alongside an online guidance intervention (Venable, 2010; Galliott, 2017). What is different, however, is that similar international studies on needs assessment (Crişan et al., 2015; Güneri et al., 2016) indicated a low preference for online counseling and low usage of Internet and other online tools for career support. In this study, however, students recognize that their preferred mode of intervention when it comes to developing career goals and discussing goal-oriented career strategies are individual online sessions and group online sessions, respectively. Moreover, the likelihood that students would use an online career service if it were provided by the Career Office of their respective universities might depend on the gender and on the specific personal career needs, with networking and soft-skills development being the top possible predictors.

There are some limitations concerning the development of this study that must be taken into consideration. First, this is an exploratory study focused on identifying current students’ career needs and whether online interventions are in demand among Portuguese higher education students. In an increasingly global academic world, it would have been important to also analyze the needs of international students who choose to pursue their studies in Portugal. Second, the sample size and the variety of students’ profiles might have been larger and more varied, but we had some difficulty in securing the collaboration of other national universities in the dissemination of the study. And finally, we had a significant drop-out rate, probably due to the length of the evaluation protocol.

Although our findings provide various insights that could be used by Career Offices to tailor their existing programs or to develop the new ones in accordance with the students’ preferences, the results on the topic of an online and computer-assisted career intervention suggest possible difficulties in using the online format for higher education students. It is interesting to note that authors state that despite obvious benefits of online career counseling (Venable, 2011; Zainuddin et al., 2020), its use is not exempt from difficulties, such as distraction problems among students (Feng et al., 2019; Chen et al., 2020; Rana et al., 2021), privacy issues (Gogus and Saygın, 2019), and frustration due to technological errors (Borko et al., 2008) or unstable connections (Rossing et al., 2012). In this regard, online and computer-assisted career guidance is more likely to be more effective if combined with face-to-face counseling (Galliott, 2017), even though providing services “with the right mix of technology and human contact” (Venable, 2010, p.94) can be a difficult goal to achieve.

## 5. Conclusion

Although much has been discussed in literature and done on international and national levels to promote the importance of career counseling for students and increase its availability, the services provision seems not to be fully developed in a large number of Portuguese HEIs with students not using the services as expected. Since there is clearly a demand for the career support that is hypothesized to increase even more, universities need to rethink the way they market career services to students and whether the websites provide easy access to all necessary information. Thus, raising awareness of the provided career services and improving the information accessibility are of great importance.

Due to the growing popularity of online career counseling (Bright, 2015) and the fact that most career offices employ a very limited number of personnel, online career interventions seem to be a perfect solution. Yet, without a meaningful consultation with service recipients, i.e., students, the creation of an online career intervention proves to be impossible. The career need assessment has a crucial role in understanding the concerns and preferences of students and translating these into action. Only in that way would the efforts and financial investments yield a service that meets students’ needs more effectively and would, in the end, be used. Although there are several advantages to online career counseling (Zainuddin et al., 2020) and most students would welcome such an intervention mode, more research is needed in order to establish possible specific concerns that could be addressed in such a format. Since there is a clear preference among students for individual career guidance, universities might need to adapt the intervention types and/or add such sessions regardless of the type.

In his historical survey, Shapin (2012, p.13) refers to universities as once being “ivory towers,” distant from the problems of the societies that created and sponsored them. It is important that Career Offices would not become such disengaged and out of reach entities, but instead would be open for the dialog with the service recipients in order to contribute to the promotion of students’ employability by adapting the range of the services and creation of targeted career guidance programs, including online interventions. In the pursuit of a better workforce, identifying student career needs, intervention preferences, and then connecting these expectations with skills development at university Career Offices, has critical implications for all parties involved—institutions, students, and prospective employers.

## Data availability statement

The raw data supporting the conclusions of this article will be made available by the authors, without undue reservation.

## Ethics statement

The studies involving human participants were reviewed and approved by CRC-W Católica Research Center for Psychological, Family, and Social Wellbeing. The patients/participants provided their written informed consent to participate in this study.

## Author contributions

All authors listed have made a substantial, direct, and intellectual contribution to the work and approved it for publication.

## Conflict of interest

The authors declare that the research was conducted in the absence of any commercial or financial relationships that could be construed as a potential conflict of interest.

## Publisher’s note

All claims expressed in this article are solely those of the authors and do not necessarily represent those of their affiliated organizations, or those of the publisher, the editors and the reviewers. Any product that may be evaluated in this article, or claim that may be made by its manufacturer, is not guaranteed or endorsed by the publisher.

## Footnotes

^1^ https://www.dgeec.mec.pt/np4/1109.html

^2^ https://ec.europa.eu/social/main.jsp?catId=1161&langId=en&intPageId=3349

^3^The assessment protocol can be retrieved here: https://ucpcienciashumanas.eu.qualtrics.com/jfe/form/SV_0195OhzUbeBOWu9?fbclid=IwAR33xUmtZgdUzIBOJ43r2ZpRoobwH9vQ5ejIvwu2fluliID8-4Axi7AgQWc

## References

[ref01] Bartik A. Bertrand M. Cullen Z. Glaeser E. Luca M. Stanton C. (2020). The impact of COVID-19 on small business outcomes and expectations. Proceedings of the National Academy of Sciences, 117, 17656–17666. doi: 10.1073/pnas.2006991117 PMC7395529 32651281

[ref4] Borko H. Jacobs J. Eiteljorg E. Pittman M. E. (2008). Video as a tool for fostering productive discussions in mathematics professional development. Teach. Teach. Educ. 24, 417–436. doi: 10.1016/j.tate.2006.11.012

[ref5] Bright J. E. (2015). If you go down to the woods today you are in for a big surprise: seeing the wood for the trees in online delivery of career guidance. Br. J. Guid. Couns. 43, 24–35. doi: 10.1080/03069885.2014.979760

[ref03] Briscoe C. S. (2002). The development and validation of an adult student’s career needs questionnaire. The University of Tennessee.

[ref6] CAEL (2018). More than just a job search. Relevant, intentional, and accessible career Services for Today’s student (and returning adults). Council for Adult and Experiential Learning Report. Available at: https://www.cael.org/hubfs/Publications/More-Than-Just-A-Job-Search-Report.pdf (Accessed November 11, 2021).

[ref7] Chen T. Peng L. Jing B. Wu C. Yang J. Cong G. (2020). The impact of the COVID-19 pandemic on user experience with online education platforms in China. Sustain. For. 12:7329. doi: 10.3390/su12187329

[ref8] Crişan C. Pavelea A. Ghimbuluţ O. (2015). A need assessment on students’ career guidance. Procedia Soc. Behav. Sci. 180, 1022–1029. doi: 10.1016/j.sbspro.2015.02.196

[ref9] Crowne K. A. Brown M. Durant D. Baburaj Y. Hornberger P. McCloskey D.. (2020). A program for embedding career activities in multiple core business courses. Int. J. Manag. Educ. 18:100421. doi: 10.1016/j.ijme.2020.100421

[ref10] Dey F. Cruzvergara C. Y. (2014). Evolution of career services in higher education. New Dir. Stud. Serv. 2014, 5–18. doi: 10.1002/ss.20105

[ref11] Feng S. Wong Y. K. Wong L. Y. Hossain L. (2019). The internet and Facebook usage on academic distraction of college students. Comput. Educ. 134, 41–49. doi: 10.1016/j.compedu.2019.02.005

[ref12] Fouad N. A. Guillen A. Harris-Hodge E. Henry C. Novakovic A. Terry S.. (2006). Need, awareness, and use of career services for college students. J. Career Assess. 14, 407–420. doi: 10.1177/1069072706288928

[ref13] Gallagher R. P. Golin A. Kelleher K. (1992). The personal, career, and learning skills needs of college students. J. Coll. Stud. Dev. 33, 301–309.

[ref14] Galliott N. Y. (2017). Online career guidance: does knowledge equate to power for high school students? J. Psychol. Couns. Sch. 27, 190–207. doi: 10.1017/jgc.2017.7

[ref15] Gallup (2016). Galup’s 2016 global great jobs report. Available at: https://www.gallup.com/services/191105/gallup-2016-global-great-jobs-report.aspx

[ref16] Giovazolias T. Leontopoulou S. Triliva S. (2010). Assessment of Greek university students’ counseling needs and attitudes: an exploratory study. Int. J. Adv. Couns. 32, 101–116. doi: 10.1007/s10447-010-9092-2

[ref17] Gogus A. Saygın Y. (2019). Privacy perception and information technology utilization of high school students. Heliyon 5:e01614. doi: 10.1016/j.heliyon.2019.e01614, PMID: 31193323 PMC6525296

[ref02] Greenhaus J. H. Callanan G. A. Godshalk V. M. (2009). Career management. Sage Publication.

[ref18] Güneri O. Owen D. W. Tanrikulu I. Dolunay F. Büyükgöze A. (2016). Examining career development needs of faculty of education students. J. Theory Pract. Educ. 17, 183–198. doi: 10.14689/ejer.2017.67.11

[ref19] Hayden S. C. Ledwith K. E. (2014). Career services in university external relations. New Dir. Stud. Serv. 2014, 81–92. doi: 10.1002/ss.20110

[ref20] IBM Corp (2019). IBM SPSS Statistics for Windows, Version 26.0. Armonk, NY: IBM Corp.

[ref04] Khurumova V. (2022). Creating targeted online career service interventions: survey of students’ career guidance needs in portuguese higher education. [Master dissertation]. Available at: https://repositorio.ucp.pt/bitstream/10400.14/37017/1/202955761.pdf

[ref21] Lairio M. Penttinen L. (2006). Students’ career concerns: challenges facing guidance providers in higher education. Int. J. Educ. Vocat. Guid. 6, 143–157. doi: 10.1007/s10775-006-9107-z

[ref22] Li X. Jung J. (2021). Career concerns and needs of mainland Chinese Master’s students in Hong Kong. Asia Pac. J. Educ., 1–15. doi: 10.1080/02188791.2021.1896477

[ref05] McBride Jr J. L. Muffo J. A. (1994). Students assess their own career goals and services needs. J. Career Planning and Employ. 54.

[ref23] NACE (2017). The class of 2017 student survey report. Available at: https://www.naceweb.org/uploadedfiles/files/2017/publication/executive-summary/2017-nace-student-survey-executive-summary.pdf (Accessed November 11, 2021)

[ref24] OECD (2019). OECD Employment Outlook 2019: The Future of Work. Paris: OECD Publishing

[ref25] Pereira M. D. de Oliveira L. C. Costa C. F. T. de Oliveira Bezerra C. M. Pereira M. D. dos Santos C. K. A.. (2020). A pandemia de COVID-19, o isolamento social, consequências na saúde mental e estratégias de enfrentamento: uma revisão integrativa. Res. Soc. Dev. 9, –e652974548. doi: 10.33448/rsd-v9i7.4548

[ref06] Pinto J. C. (2019). Psychological counseling in Portuguese higher education: what are the students’ needs? Universitas Psychologica 18, 1–15.

[ref07] Pinto J. C. Faria L. Pinto H. R. Taveira M. D. C. (2016). Identificação de necessidades de intervenção psicológica: um estudo-piloto no ensino superior português [Assessment of psychological intervention needs: a pilot study at the Portuguese higher eeducation] Psicologia USP 27, 459–472.

[ref08] Pinto J. C. Taveira M. C. (2010). Seminário de gestão pessoal da carreira: avaliação de um programa de intervenção psicológica no ensino superior [Self-career management seminar: assessment of a psychological intervention program at higher education]. Oral communication presented at I Seminário Internacional – Contributos da Psicologia em Contextos Educativos. Braga: Minho’s University.

[ref26] Rana S. Garbuja C. K. Rai G. (2021). Nursing students’ perception of online learning amidst COVID-19 pandemic. J. Lumbini Med. College 9:6. doi: 10.22502/jlmc.v9i1.408

[ref27] Rossing J. P. Miller W. Cecil A. K. Stamper S. E. (2012). iLearning: the future of higher education? Student perceptions on learning with mobile tablets. J. Scholar. Teach. Learn. 12, 1–26.

[ref28] Shapin S. (2012). The ivory tower: the history of a figure of speech and its cultural uses. Br. J. Hist. Sci. 45, 1–27. doi: 10.1017/S0007087412000118

[ref29] Stolzenberg E. B. Eagan M. K. Aragon M. C. Cesar-Davis N. M. Jacobo S. Couch V.. (2019). The American Freshman: National Norms Fall 2017. Los Angeles: Higher Education Research Institute, University of California.

[ref30] U.S. Department of Education (2020). National Center for education statistics. The condition of education 2020 (NCES 2020–144). Employment and Unemployment Rates by Educational Attainment.

[ref31] Venable M. (2010). Using technology to deliver career development services: supporting Today's students in higher education. Career Dev. Q. 59, 87–96. doi: 10.1002/j.2161-0045.2010.tb00132.x

[ref32] Venable M. (2011). What influences your career choice. Available at: https://www.onlinecollege.org/about-us/ (Accessed March 11, 2016).

[ref33] Vinson B. M. Reardon R. C. Bertoch S. C. (2014). Career services at colleges and universities: a 30-year replication study. J. Coll. Stud. Dev. 55, 203–207. doi: 10.1353/cs.2014.0018

[ref34] Yang M. Y. You M. (2010). A survey of career guidance needs of industrial design students in Taiwanese universities. Asia Pac. Educ. Rev. 11, 597–608. doi: 10.1007/s12564-010-9106-0

[ref35] Zainuddin Z. Chu S. K. W. Shujahat M. Perera C. J. (2020). The impact of gamification on learning and instruction: a systematic review of empirical evidence. Educ. Res. Rev. 30:100326. doi: 10.1016/j.edurev.2020.100326

